# Effects and Mechanism of Blue Light on *Monascus* in Liquid Fermentation

**DOI:** 10.3390/molecules22030385

**Published:** 2017-03-01

**Authors:** Xiaowei Zhang, Wenqing Liu, Xiying Chen, Junhui Cai, Changlu Wang, Weiwei He

**Affiliations:** 1 College of Food Science & Engineering, Xuchang University, Xuchang 461000, China; xiaoweizhang1982@163.com (X.Z.); m13298265891@163.com (W.L.); m15993640173@163.com (X.C.); 2 Key Laboratory of Micro-Nano Materials for Energy Storage and Conversion of Henan Province, College of Advanced Materials and Energy, Institute of Surface Micro and Nano Materials, Xuchang University, Xuchang 461000, China; cjhyinian@163.com; 3 Department of Food Biotechnology, Tianjin University of Science and Technology, 1038 Dagu South Road, Hexi District, Tianjin 300222, China

**Keywords:** *Monascus*, catalase, blue light, citrinin yield, photodegradation

## Abstract

The effect of light on *Monascus* and the underlying mechanism have received a great deal of interest for the industrial application of *Monascus* pigments. In this study, we have examined the effects of blue light on the culture morphology, mycelium growth, pigments, and citrinin yield of *Monascus* in liquid-state and oscillation fermentation, and explored the mechanism at a physiological level. It was found that blue light affected the colony morphology, the composition (chitin content), and permeability of the *Monascus* mycelium cell wall in static liquid culture, which indicates blue light benefits pigments secreting from aerial mycelium to culture medium. In liquid oscillation fermentation, the yields of *Monascus* pigments in fermentation broth (darkness 1741 U/g, blue light 2206 U/g) and mycelium (darkness 2442 U/g, blue light 1900 U/g) cultured under blue light and darkness are different. The total pigments produced per gram of *Monascus* mycelium under blue light was also higher (4663 U/g) than that in darkness (4352 U/g). However, the production of citrinin (88 μg/g) under blue light was evidently lower than that in darkness (150 μg/g). According to the degradation of citrinin caused by blue light and hydrogen peroxide, it can be concluded that blue light could degrade citrinin and inhibit the catalase activity of *Monascus* mycelium, subsequently suppressing the decomposition of hydrogen peroxide, which is the active species that degrades citrinin.

## 1. Introduction

As a natural food colorant, *Monascus* pigments have been applied for thousands of years in China and have potential for wide industrial applications [1,2]. Multiple studies have reported that *Monascus* not only produces pigments that can improve sensory characteristics of food, but also generates many secondary metabolites with physiological activities such as anti-hyperlipidemia [3], antioxidant [4], anti-inflammatory [5], antihypertension [6], antibiosis [7], memory improvement [8,9], and so on. *Monascus* is widely utilized as a food additive in 20 kinds of food without any adverse health effects [1,10]. However, scientists have reported that citrinin, which is toxic to human kidney and liver, is widespread in *Monascus* [11,12]. Therefore, approaches for reducing the level of citrinin in *Monascus* products have become an important task for *Monascus* researchers. Yang et al. [13] found that with a three-stage aeration process with shifting oxygen supply in a 10 L fermentor, pigment production was increased by 29.6% and citrinin concentration was reduced by 79.5% compared to constant one-stage cultivation. Hajjaj et al. [14] showed that adding different amino acids (especially histidine) used as sole nitrogen sources to liquid medium has a significant impact on the production of water-soluble red pigments and citrinin in fermentation products. Furthermore, Haggblom et al. [15] reported that blue light inhibited alternariol and alternariol monomethyl ether production by 69% and 77%, respectively. Fanelli et al. [16] summarized recent findings on the effect of specific light wavelength and intensity on mycotoxin biosynthesis in the main toxigenic fungal genera. Fanelli et al. [17] also found the influence of different light wavelengths on the biosynthesis of ophiobolin A by *Bipolaris maydis* are different; white light allowed the highest production of the metabolite, but blue and green light showed an inhibitory effect, reducing the production to 50%. As an important environmental factor, light, specifically the shorter-wavelength blue light, plays a key role in physical processes of many fungi [18], including entrainment and resetting of circadian clock, biosynthesis of photoprotective pigments, induction of asexual conidiospores, and so on. As an important strain in industrial production [19], it is known that the colony morphology, spore germination, sporulation, pigment yield, and distribution of *Monascus* are sensitive to light. Bühler et al. [20] found light intensity is an important factor that can inhibit the growth and pigment production of *Monascus ruber,* and growth and pigment production were higher in Petri dishes and flasks exposed to red light and in the absence of light. Chen et al. [21] proved excessive blue light (constant exposure to blue light of 100 lux) reduced the yields of six pigments in mycelium, but an appropriate illumination level of blue light (exposure to 100 lux of blue light once for 30 min/day and to 100 lux of blue light once and twice for 15 min/day) could increase production of some pigments, and affected the growth as well as the expression levels of pigment biosynthetic genes in *Monascus*. All studies have proved light (especially blue light) has an apparent effect on *Monascus* growth, production of metabolites (pigments, monacolin K, citrinin, and so on), and the expression of related genes. It is well known that the yield of metabolites is not only determined by the expression of related genes, but is also related to the composition of cell, enzyme activity, and so on. However, there are few reports concerning the effect of blue light on cell composition and stability of citrinin. In this work, we have employed liquid-state fermentation for studying the effects of continuous blue light on mycelium growth, pigments, citrinin content, and the composition of *Monascus* mycelium wall, and we discuss the influence and mechanism of blue light on *Monascus* citrinin yield. Our findings will provide insights into future studies of fungal growth and metabolite production in blue light conditions. Furthermore, our study investigated the main reason that the yield of pigments and citrinin are different when exposed to blue light and darkness, and our results will provide evidence for technical and theoretical application of optically controlled fermentation for raising yield of *Monascus* pigments and decreasing yield of citrinin.

## 2. Results and Discussion

### 2.1. Effect of Blue Light on Monascus Colony Phenotype in Static Liquid Culture

**Figure 1:**
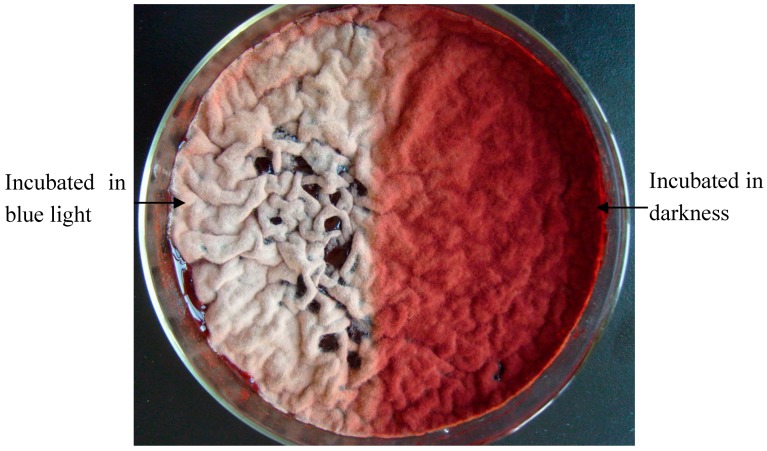
Effect of blue light on *Monascus* culture morphology in liquid fermentation. Left part and right part of the culture dish were separately exposed to either blue light or protected by tinfoil paper for 5 days.

Half of the *Monascus* culture was covered (dark conditions) with tinfoil paper for comparison, and the other half was continuously cultured under blue light for 5 days at 30 °C, as described in Section 3.3. The colony phenotype of the culture exposed to blue light for 5 days is shown in Figure 1.

**Figure 2:**
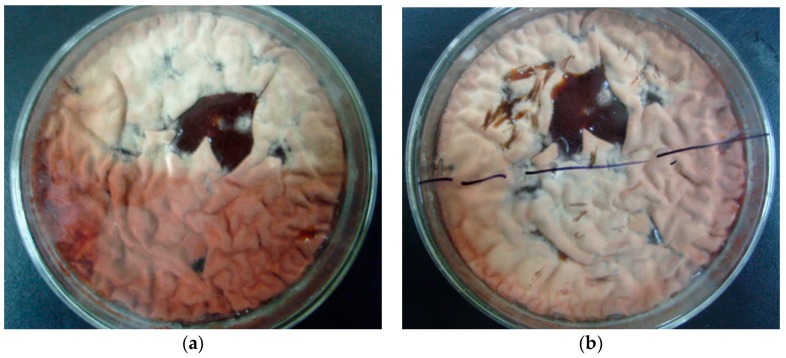
Changes in *Monascus* culture morphology after exposure to blue light. (**a**) Culture morphology of *Monascus* cultured under blue light for 5 days with tinfoil paper covering the bottom part; (**b**) Morphology of the culture in (**a**) exposed to blue light for additional 8 h after removing the tinfoil paper.

In static liquid culture, *Monascus* pellicles appeared gray and deep-red under blue light and in darkness, respectively. This suggested that *Monascus* pellicles treated with blue light had lower pigment content than those kept in darkness. It also implied that blue light might have a great influence on *Monascus* phenotype. Figure 2 shows the colony phenotype of the culture before and after removal of tinfoil paper on day 5, and additional exposure to blue light for 8 h.

Figure 2 shows that after blue light exposure for 8 h, the deep-red pellicle that was previous grown in darkness presented the same phenotype as those that were continuously cultured in blue light. This suggested that *Monascus* is sensitive to the presence of blue light, and can adapt to the blue-light environment by adjusting its pigment content in the pellicle in order to satisfy its own survival needs.

### 2.2. Possible Explanation for the Reduction in Monascus Pellicle Pigment Content after Blue Light Exposure

To find out the reasons for blue light affecting the culture morphology of *Monascus*, four of the pellicles, which appeared deep-red after 5 days of culturing in darkness, were selected. Three of these pellicles were tiled onto three pieces of clean glass; one of them received no treatment while the others were sterilized under high-pressure steam to inactivate *Monascus*. One inactivated *Monascus* pellicle was transferred onto a fresh solid flat surface, and the other remained on the clean glass. The fourth selected pellicle was transferred onto a fresh solid flat surface in order to preserve its physiological activities. We found that the four pellicles were exposed to blue light irradiation for 8 h, the fourth pellicle, having physiological activities, showed the same pale appearance as pellicles cultured in continuous blue light. Although the pellicle that did not receive sterilization was less vibrant than pellicles cultured in darkness, it was still deep-red in color after exposure to blue light for 8 h. On the other hand, sterilized pellicles on glass and fresh solid flat surfaces showed minor differences in pigment color before and after blue light exposure. These phenomena are displayed in Section 2.1. Our results suggested that the reduction of *Monascus* pigment content after blue-light irradiation could mainly be attributed to the physiological activities of *Monascus* [20,22], rather than the photodegradative effect of blue light on *Monascus* pigments. Our findings also supported that blue-light receptors are present in *Monascus* (WC-1 and WC-2 have been reported as blue-light receptors in fungi [20,21]). It is possible that the induction and signal transduction through blue-light receptors in *Monascus* could be responsible for the difference in responses of *Monascus* cultured in various light conditions.

### 2.3. Effect of Blue Light on Monascus Mycelium Permeability

**Figure 3:**
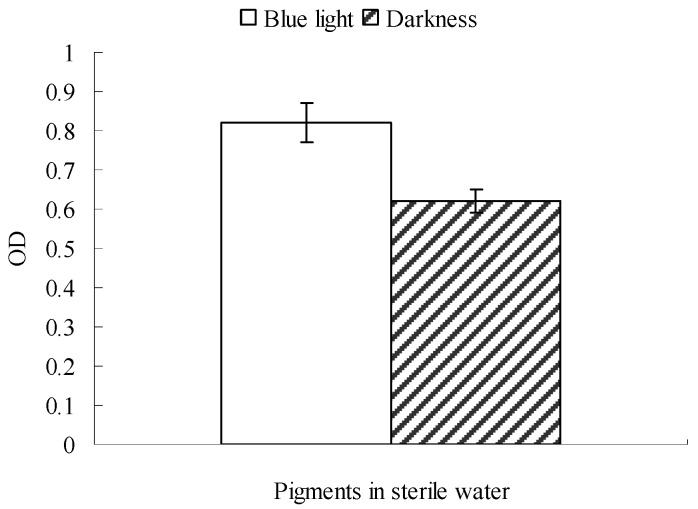
Effect of blue light on resting cell permeability of *Monascus.* Six resting pellicles (with the same pigment production) were respectively spread on six culture dishes containing 10 mL sterile water. Three pellicles were irradiated with blue light (0.16 mW/cm^2^) for 8 h while the others were kept in darkness. The absorbance at 510 nm of the distilled water was determined after 8 h.

Results described in Section 2.1 and Section 2.2 indicate that blue light could affect *Monascus* physiological activities. Mycelium membrane permeability is an important physiological activity, so it is very necessary to discuss the effect of blue light on *Monascus* mycelium permeability. The effect of blue light on *Monascus* resting membrane permeability, performed as described in Section 3.4, is shown in Figure 3. Figure 3 shows the absorbance at 510 nm of sterile water in blue light was 0.82, which was significantly higher than the absorbance of 0.62 obtained from sterile water in darkness. This showed that *Monascus* pigment content in sterile water was higher under blue light. The *Monascus* pigments in sterile water could only be derived from the pellicle since nutrient was almost entirely absent in sterile water, which prevented any *Monascus* growth. Our results suggested that blue light enhances release of *Monascus* pigments from pellicles to the surrounding environment. In addition, mycelium cell permeability of *Monascus* was also improved by exposure to blue light.

### 2.4. Effect of Blue Light on the Composition of Monascus Mycelium Wall

**Figure 4:**
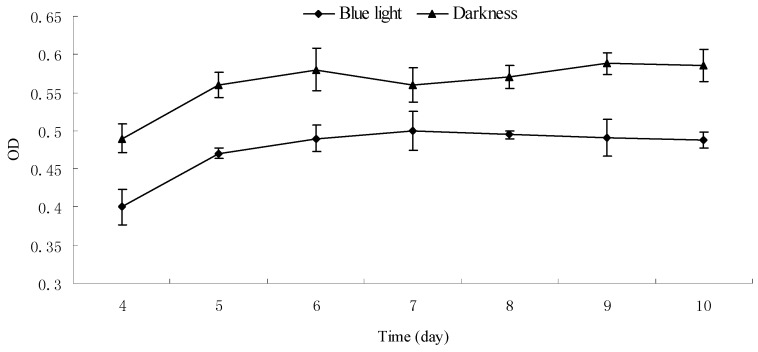
Effect of blue light on glucosamine content of *Monascus* mycelium wall. *Monascus* mycelium wall is composed of chitin, whose content is determined by the measurement of glucosamine released by the acid hydrolysis of chitin. The amount of glucosamine in *Monascus* mycelium wall cultured under blue light and darkness was determined by the absorbance at 530 nm. Experimental conditions are described in Section 3.9.

It was established that blue light had an effect on *Monascus* mycelium permeability, as discussed in Section 2.3. However, mycelium permeability is related to the composition of *Monascus* mycelium wall. Therefore, it is necessary to discuss the effect of blue light on the composition of *Monascus* mycelium wall. Dry *Monascus* mycelia that were cultured for 4–10 days in darkness and under continuous blue light were selected, respectively. In general, fungi mycelium wall is mainly composed of chitin, whose content is determined by the measurement of glucosamine [23]. Glucosamine content in the *Monascus* mycelia was determined according to the method described in Section 3.9. Results are shown in Figure 4. As revealed in Figure 4, glucosamine content in *Monascus* mycelium from cultures under blue light was lower than those in darkness throughout the entire culture period. This indicated that blue light could affect chitin content in the *Monascus* mycelium cell wall, which could further influence the permeability of the *Monascus* mycelium cell, thereby enhancing *Monascus* pigment secretion from aerial hyphae to culture medium. These observations were consistent with the measurements of pigment content in sterile water after subsequent blue light irradiation.

### 2.5. Effect of Blue Light on the Growth and Pigment Yield of Monascus Cultured by Liquid Oscillation

**Figure 5:**
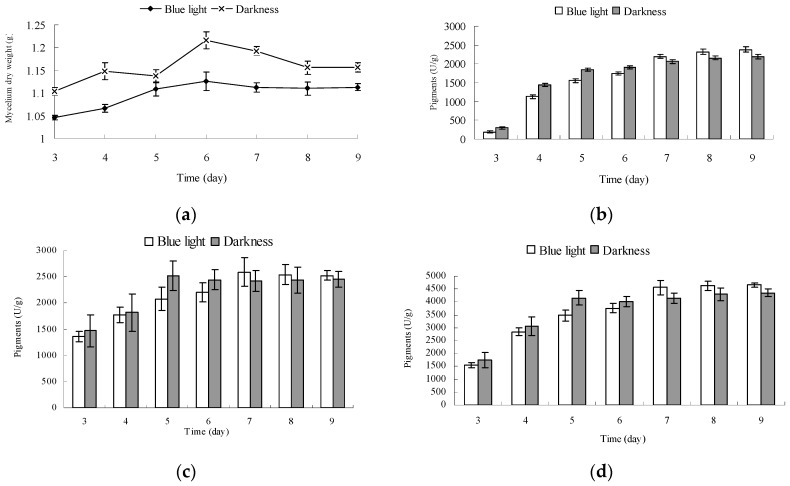
Effect of blue light on *Monascus* mycelium weight and Monascus pigment production. (**a**) Effect of blue light on the weight of *Monascus* mycelium; (**b**–**d**) Effect of blue light on Monascus pigment production in fermentation broth and *Monascus* mycelium, and total pigment production, respectively. *Monascus* was cultured by liquid oscillatory fermentation at 30 °C, 180 r/min for 9 days under blue light (0.16 mW/cm^2^) and in darkness. The growth of mycelium and the yield of *Monascus* pigment production under blue light and in darkness were respectively determined by the mycelial dry weight and absorption at 510 nm.

Dry *Monascus* mycelium weight and pigment production in fermentation broth and mycelium of *Monascus* cultured under blue light and in darkness were determined. The results are shown in Figure 5.

**Figure 6:**
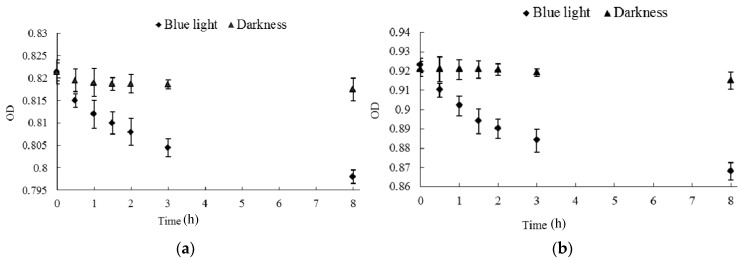
Effect of blue light on the stability of *Monascus* pigments in fermentation broth and mycelium. (**a**) The stability of Monascus pigments in fermentation broth; (**b**) the stability of Monascus pigments in hyphae. Monascus pigments in fermentation broth and mycelium ethanol extraction were randomly assigned to two groups of three samples. One group was exposed to blue light while the other group was kept in darkness. Absorbance at 510 nm of pigments’ solution was determined every 20 min, and the average absorbance of three samples was calculated.

The mycelium dry weight of *Monascus* cultured for 6 days under blue light and in darkness reached a maximum value of 1.126 g and 1.216 g, respectively (Figure 5a). The mycelium dry weight of *Monascus* cultured under blue light was fairly stable after 6 days, however, that of *Monascus* cultured in darkness decreased noticeably. The mycelium dry weight of *Monascus* cultured under blue light was lower than that of those cultured in darkness throughout the entire culture process. This could be attributed to the fact that blue light is not conducive to the growth of *Monascus*, which is consistent with the results of Chen [21]. In the early stage of fermentation (3–6 day), the pigment contents in fermentation broth and mycelium under blue light were less than those in darkness (Figure 5b–d). This could be explained by blue light leading to a certain amount of degradation of *Monascus* pigments, as shown in Figure 6. In this early stage, synthesis of *Monascus* pigments is less substantial, therefore, any decomposition caused by blue light would be more obvious. When mycelium weight reached the maximum on day 6, pigments in fermentation broth under blue light increased significantly. The pigment contents of *Monascus* in fermentation broth grown under blue light for on days 6 and 7 were, respectively, 1741 U/g and 2194 U/g. The increase in pigment content in fermentation broth in darkness was not as noticeable as that exposed to blue light. The pigment yields of *Monascus* in fermentation broth grown in darkness on days and 7 were, respectively, 1900 U/g and 2060 U/g. After 6 days, *Monascus* pigment yield in mycelium cultured under blue light continued to increase; however, the pigment content in mycelium cultured in darkness remained fairly unchanged. The pigment contents in *Monascus* mycelium incubated under blue light and darkness for 7 days were 2593 U/g and 2417 U/g, respectively. Results in Section 2.4 showed that chitin content of mycelium wall of *Monascus* cultured under blue light was less than that in darkness, which would influence the permeability of *Monascus* mycelium cell, enhancing *Monascus* pigment secretion from aerial hyphae to culture medium. Therefore, it is likely that pigments within the mycelium cultured under blue light were more easily transported from the inside to the outside of the mycelium than those cultured in darkness. This would also explain the noticeable increase in pigment content of fermentation broth cultured under blue light after 6 days. Due to the decrease in feedback inhibition of *Monascus* pigment synthesis under blue light, pigment production in mycelium continued to increase, and pigment yield per unit mycelium weight was higher than that cultured in darkness. The total pigment yields produced by a unit mycelium weight cultured under blue light and in darkness after 9 days were, respectively, 4663 U/g and 4352 U/g.

### 2.6. Influence and Mechanism of Blue Light on Monascus Citrinin Yield in Liquid-State Fermentation

**Figure 7:**
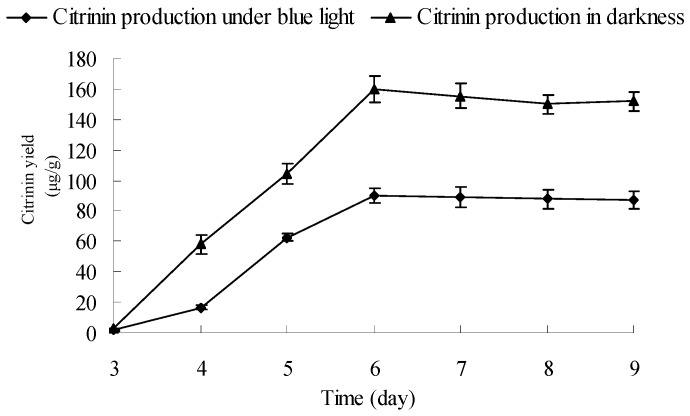
Effect of blue light on citrinin production. Experimental conditions are described in Section 3.8. *Monascus* was cultured by liquid oscillatory fermentation at 30 °C, 180 r/min for 9 days under blue light (0.16 mW/cm^2^) and in darkness. The citrinin production in different culture condition were determined by high-performance liquid chromatograph.

To investigate the influence of blue light on *Monascus* citrinin yield, the citrinin yields of *Monascus* cultured by liquid-state fermentation under blue light and in darkness were dynamically measured. Results are shown in Figure 7. Figure 7 shows that citrinin yields of *Monascus* cultured by liquid-state fermentation under blue light and darkness simultaneously reached maximum levels of 90 μg/g and 160 μg/g, respectively. In the course of cultivation (3–9 days), the citrinin yield under blue light was lower than in darkness. At the end of fermentation (9 days), the citrinin yields of *Monascus* under blue light and darkness were, respectively, 88 μg/g and 150 μg/g. During the entire liquid-state fermentation process, the weight of *Monascus* mycelium under the blue-light culture condition was lower than that in darkness; however, the difference in mycelium weight was not the ultimate reason that led to a 50% decrease of final citrinin yields between *Monascus* cultured under the two conditions. In fact, the degradation of citrinin caused by blue light in fermentation broth could be one attributing factor. In addition, *Monascus* contains light photoreceptors that allow for blue light inductive signaling, which, in turn, leads to changes of *Monascus* intracellular metabolic pathways that ultimately affect the synthesis of enzymes that degrade citrinin or inhibit the synthesis of citrinin in *Monascus*. In order to investigate the mechanism that contributes to the decrease in citrinin content under blue light irradiation during *Monascus* liquid-state fermentation, we performed the following experiments.

### 2.7. Effect of Blue Light on Citrinin Degradation

**Figure 8:**
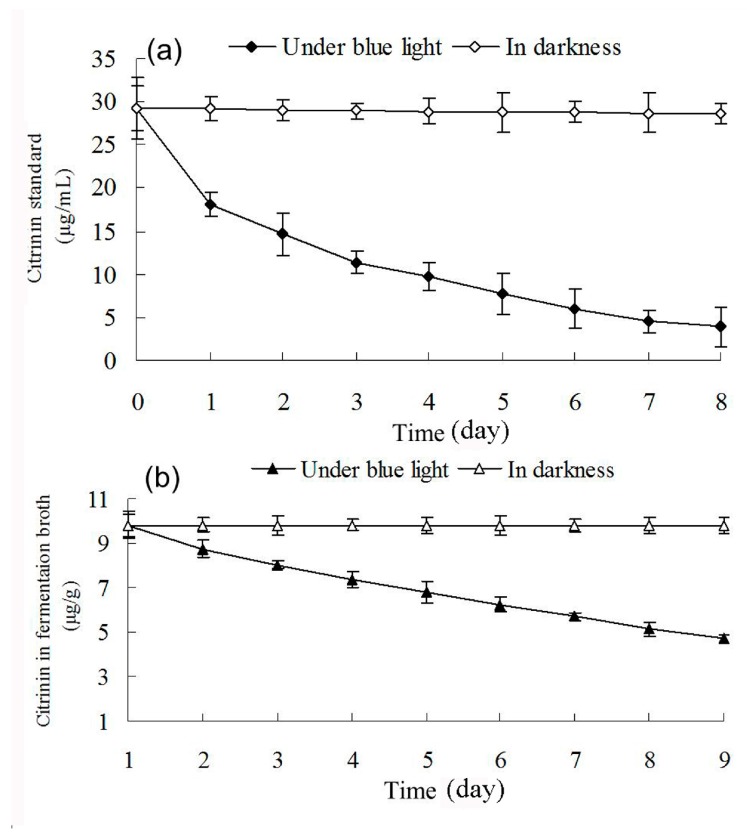
Effects of blue light on the stabilities of citrinin standard (**a**) and citrinin in fermentation broth (**b**). Citrinin standard methanol solution and citrinin of *Monascus* fermentation ethanol extraction were respectively exposed to blue light and kept in darkness. Citrinin content was determined by HPLC at specific time intervals.

Changes in citrinin content in methanol solution containing citrinin standard and in ethanol extraction containing *Monascus* liquid fermentation broth were dynamically examined under blue light irradiation and in darkness. Figure 8 shows that blue light affects degradation of citrinin in both methanol solution with citrinin standard and in ethanol extraction with liquid fermentation of *Monascus*. This can mainly be attributed to the presence of conjugated bonds in the citrinin molecule, which leads to electron transition after absorbing short-wavelength photons such as blue light. The electron transition disrupts the molecular structure of citrinin and leads to degradation of citrinin [23]. The concentration of the citrinin standard in methanol solution decreased from 29.24 μg/mL to 3.88 μg/mL after 8 days of blue-light irradiation, while the citrinin concentration of *Monascus* in the liquid fermentation product reduced from 9.81 μg/mL to 5.29 μg/mL. Based on these observations, blue light appeared to have a stronger effect on the degradation of citrinin standard. The lesser effect of blue light on degradation of *Monascus* fermentation broth may be due to the presence of a variety of pigments, especially red pigments, in the fermentation liquid, which absorbed blue light and resulted in a decrease in blue-light permeability. The intensity of citrinin absorption of blue light in fermentation broth was also greatly weakened; therefore, the effect of blue light on citrinin degradation in fermentation broth was less drastic than that on citrinin standard solution. Our results demonstrated that the effect of blue light on citrinin degradation is one of the important reasons for the decrease in citrinin content in fermentation broth under blue light. This is consistent with the results of Schmidt-Heydt [24].

### 2.8. Effect of Hydrogen Peroxide on the Stability of Citrinin

**Figure 9:**
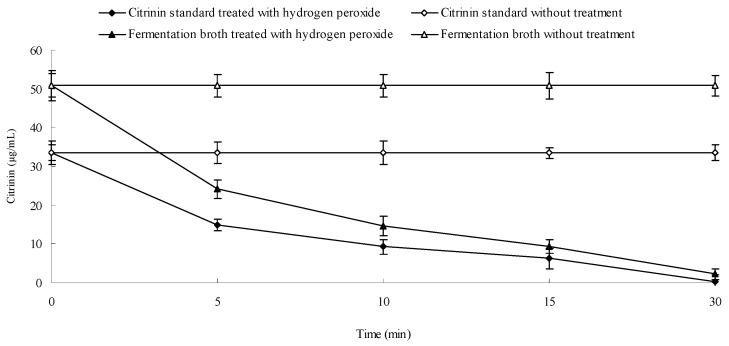
Effect of H_2_O_2_ on citrinin stability. Hydrogen peroxide solution (0.05 mL, 30%) was respectively added to 3.6 mL citrinin standard methanol solution and citrinin extraction solution of *Monascus* fermentation broth. Citrinin content was determined by HPLC at regular time intervals.

Apart from blue light, hydrogen peroxide can also degrade citrinin. Changes in citrinin content were dynamically measured after the addition of 0.05% hydrogen peroxide to methanol solution with citrinin standard and to liquid fermentation broth. Results are shown in Figure 9. Figure 9 shows that the concentration of citrinin standard reduced from 33.49 μg/mL to 0.31 μg/mL 30 min after addition of 0.05% H_2_O_2_. Citrinin content in *Monascus* fermentation broth also decreased from 50.83 to 2.15 µg/mL after addition of 0.05% H_2_O_2_. Our results demonstrated that H_2_O_2_ has a strong effect on citrinin degradation in both the fermentation liquid and in standard methanol solution, which has been reported by Hajjaj [25]. H_2_O_2_ is a harmful product present in aerobic eukaryotes. It is possible that H_2_O_2_ content in *Monascus* mycelium under blue light was higher than that in darkness, which could potentially explain the lower citrinin content in fermentation liquid under blue light than that in darkness. H_2_O_2_ content in organisms is often associated with the presence of peroxidase and catalase activity. We examined catalase activity in *Monascus* mycelium under blue light and in darkness in order to better understand the effect of H_2_O_2_ content on citrinin stability.

### 2.9. Effect of Blue Light on Catalase Activity in Monascus Mycelium in Liquid Fermentation

**Figure 10:**
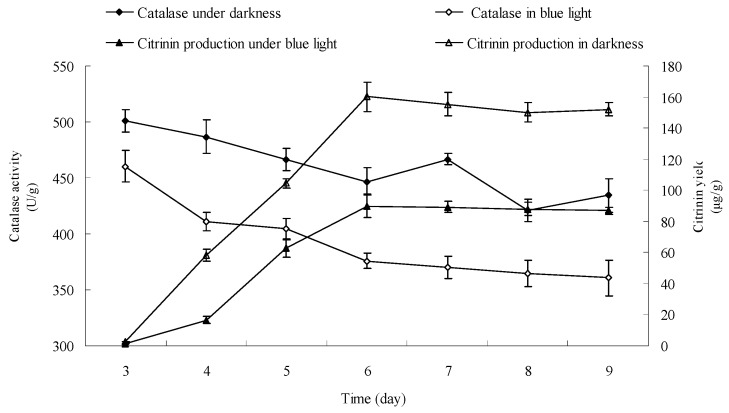
Effect of blue light on catalase activity of *Monascus* in liquid fermentation. Two grams of wet *Monascus* mycelium treated with blue light or kept in darkness were added to 18 mL 0.2 mol·mL^−1^, pH 7.8 phosphate buffer from fermentation of day 3. Catalase activity of 10% tissue homogenate was determined by Catalase Assay Kit (Sigma-Aldrich Corporation, St. Louis, MO, USA).

Catalase activity in *Monascus* mycelium was dynamically examined from day 3 to day 9 according to the method described in Section 3.13. Results are shown in Figure 10. Figure 10 shows that the catalase activities in *Monascus* mycelium were 460.45 U/g and 501 U/g, respectively, in early fermentation (day 3) under blue light and in darkness. The corresponding citrinin yields were 1.5 μg/g and 2.73 μg/g, respectively. The catalase activities were 361 U/g and 434 U/g and the corresponding citrinin yields were 88 μg/g and 152 μg/g at the end of fermentation (day 9). The catalase activity in *Monascus* mycelium and the citrinin yield under blue light were both lower than those in darkness throughout the entire fermentation process. Figure 9 showed that H_2_O_2_ had a strong effect on degradation of citrinin. H_2_O_2_ content is closely linked to the activity of catalase. Catalase catalyzes the transformation of H_2_O_2_ into nontoxic water and oxygen, therefore, the level of catalase activity directly predicts the amount of H_2_O_2_ within *Monascus* mycelium. Catalase activity in *Monascus* mycelium under blue light was lower than that in darkness, which could result in an increase of H_2_O_2_ accumulation, which is consistent with a previous report [26]. The presence of H_2_O_2_ might have a destructive effect on citrinin, which would explain the lower citrinin content under blue light than that in darkness.

## 3. Materials and Methods

### 3.1. Strain and Culture

Culture of *Monascus ruber* N, a high pigment-producing strain (offered by Professor Changlu Wang, Tianjin University of Science and Technology, Tianjin, China), was used in this study. It was maintained on malt wort extract agar slant, cultured at 30 °C for 7–9 days, then preserved at 4 °C and subcultured once every 3 weeks.

### 3.2. Inoculum Preparation

Five milliliters of sterile distilled water was added to a fresh fully sporulated agar slope culture. Then, the spores were scraped under strict aseptic conditions. The spore suspension was inoculated in 50 mL seed medium (3% rice flour, 0.25% KH_2_PO_4_, 0.2% NaNO_3_, 0.1% MgSO_4_•7H_2_O. The initial pH of the medium was adjusted to 4.5 with lactic acid) in a 250 mL flask. Then, the flask was incubated at 30 °C at 180 r/min for 30 h. The culture was then filtered through sterile glass wool, and the spore suspension containing unclumped spores with no hyphae was adjusted to 2 × 10^7^ spores per mL. The obtained spore suspension was used as inoculum.

### 3.3. Stationary Liquid Culture of Monascus

Fifty milliliters of sterile liquid fermentation medium (rice powder 100 g/L, KH_2_PO_4_ 1.5 g/L, NaNO_3_ 3 g/L, and MgSO_4_•7H_2_O 1 g/L) was added to a culture dish of 20 cm in diameter. Three milliliters of the inoculum at concentration of 2 × 10^7^ spores per mL was also added. The culture was thoroughly mixed, and allowed to grow under blue light and darkness, respectively, for 8 days at 30 °C in a constant-temperature light growth chamber. Specifically, Petri dishes containing seed fermentation broth were randomly assigned into two groups. With all other parameters the same, one group was cultured in a constant-temperature incubator with a 1 W blue LED monochromatic lamp (constant biochemical incubator, LRH-250CB, Shanghai Heng Yi Scientific Instrument Co., Ltd., Shanghai, China), while the other one was cultured in darkness for 8 days.

### 3.4. Effect of Blue Light on Monascus Resting Pellicle Permeability

Six parallel *Monascus* pellicles (with the same pigment production), cultured as described in Section 3.3, were picked out after culturing for 6 days in darkness. The *Monascus* resting pellicles were rinsed with sterile water to properly remove the extracellular *Monascus* pigments. Then, the six resting pellicles were respectively spread on six culture dishes containing 10 mL sterile water. The Petri dishes were randomly assigned to two groups of three samples. One group was irradiated with blue light (0.16 mW/cm^2^) for more than 8 h while the other group was kept in darkness. Finally, the *Monascus* pigment content in the sterile water culture was determined after 8 h.

### 3.5. Oscillatory Liquid Fermentation of Monascus

Three milliliters of seed suspension at 2 × 10^7^ spores/mL were added to the 250 mL triangular flask containing 50 mL of fermentation liquid. The inoculated fermentation medium was cultured in a constant-temperature light growth chamber at 30 °C at 180 r/min for 9 days under blue light (0.16 mW/cm^2^) and in darkness. The specific experimental condition was the same as described in Section 3.3.

Three days after fermentation, three samples from each group were randomly examined every 24 h. Dynamic monitoring was performed to determine the dry weight of mycelium, yield of *Monascus* pigments, and citrinin production under different conditions in order to determine the effects and mechanism of blue light on *Monascus* growth, as well as pigment and citrinin contents in *Monascus* fermentation broth and *Monascus* mycelium.

### 3.6. Dynamic Monitoring of Dry Weight of Mycelium during Liquid Fermentation

The fermentation liquid sample was placed in a centrifuge cup and centrifuged at 5000 r/min. Pigment content in upper supernatant broth was determined according to methods described in Section 3.7. Mycelium in the precipitation was rinsed with distilled water and then centrifuged. The supernatant was then discarded and the whole process was repeated three times. The mycelium was dried in a vacuum oven at 60 °C and then weighed to determine the mycelial dry weight of *Monascus*.

### 3.7. Determination of Monascus Pigment Yield in Liquid Fermentation

#### 3.7.1. Determination of Pigment Yield in Fermentation Broth

Pigments produced by the *Monascus* fermentation process are a mixture of three colors (red, orange, and yellow), which have maximum absorption peaks at 510 nm, 465 nm, and 410 nm, respectively. Pigment contents in fermentation broth can be determined at the corresponding wavelengths by ultraviolet–visible spectrophotometer. The absorbance A_410_, A_465_, and A_5__10_ were used to calculate the yield of each of the three colored pigments: S_410_, S_465_, and S_510_ represent the color values of *Monascus* yellow pigments, orange pigments, and red pigments, respectively. Changes in production of red pigments were mainly reviewed in this experiment (1).

(1) $$S=\frac{A\times n}{G}$$

In this formula, *S* is the color value of fermentation broth; *G* is the dry weight of *Monascus* mycelium; n is the dilution ratio.

#### 3.7.2. Determination of Pigment Yield in *Monascus* Mycelium

To determine the pigment content in *Monascus* mycelium, 150 mL 80% ethanol solution was added to a 250 mL triangular flask containing 5 g *Monascus* mycelium. The mixture was then treated by ultrasonic wave for 30 min, oscillated at 180 r/min for 24 h, and then centrifuged at 5000 r/min for 20 min. Pigment yield was calculated according to method described in Section 3.7.1.

#### 3.7.3. Determination of Total Pigment Yield in Unit Mycelium Dry Weight

Total pigment yield in unit mycelium = pigment yield in fermentation broth + pigment yield in mycelium.

### 3.8. Determination of Citrinin Yield

#### 3.8.1. Pretreatment of *Monascus* Sample

Two times volume of 80% ethanol solution was added to a certain volume of *Monascus* fermentation broth, mixed well, incubated in water bath for 1 h at 60 °C, oscillated every 20 min, and centrifuged at 5000 r/min for 20 min. Then, the supernatant was filtered through a 0.45 µm Millipore filter.

#### 3.8.2. Condition of HPLC

LC-10ATVP high-performance liquid chromatography was performed as follows: column Shimadzu VP-ODS C18 (5 μm, 250 mm × 4.6 mm), temperature 30 °C, injection volume 20 mL, flow rate 1.0 mL/min, and the mobile phase acetonitrile:methanol:water (pH 2.5) = 70:10:20. Fluorescence detection: λ_ex_ = 331 nm, λ_em_ = 500 nm.

### 3.9. Determination of *Monascus* Mycelium Wall Component

*Monascus* mycelium mainly consists of chitin. Chitin content of *Monascus* mycelium wall was evaluated by glucosamine measurement. Dry mycelium cultured under blue light and in darkness for 5–9 days was weighed. One milliliter of 60% sulfuric acid solution was added to 0.15 g of dry mycelium, and the mixture was soaked for 24 h at 25 °C, diluted to 1 mol/L^−1^ in a 50 mL volumetric flask, heated for 20 min under high pressure and cooled, then adjusted pH to 7.0 with 1 mol/L^−1^ NaOH. Subsequently, 1 mL acetyl acetone reagent (3.5 mL acetyl acetone + 50 mL 1.2 mol/L^−1^ Na_2_CO_3_) was added to a 2 mL sample (2 mL distilled water as control), and then heated for 30 min in a boiling water bath. Two milliliters of absolute ethanol and 1 mL *p*-dimethylaminobenzaldehyde reagent (1.333 g *p*-dimethylaminobenzaldehyde dissolved in 25 mL absolute ethanol and concentrated hydrochloric acid in brown bottle) were added, and the sample was oscillated, followed by addition of 4 mL absolute ethyl alcohol and thermal insulation for 1 h at 60 °C, prior to determination of absorbance at 530 nm [23].

### 3.10. Effects of Blue Light on Monascus Pigments Stability

Sixty milliliters of *Monascus* pigments in ethanol solution were divided into six equal parts at an absorbance value of 0.87 at 510 nm. The six parts were randomly assigned to two groups of three. One group was exposed to blue light while the other group was kept in darkness. Absorbance was determined every 20 min, and the average absorbance was calculated.

### 3.11. Effects of Blue Light on Citrinin Stability

A specific volume of citrinin standard methanol solution and *Monascus* fermentation broth were respectively exposed to blue light and kept in darkness. Citrinin content was determined by HPLC at specific time intervals.

### 3.12. Effect of Hydrogen Peroxide on Citrinin Stability

To determine the effects of hydrogen peroxide on citrinin stability, 0.05 mL of 30% hydrogen peroxide solution was respectively added to 3.6 mL citrinin standard methanol solution and citrinin extraction solution of *Monascus* fermentation broth. Citrinin content was determined by HPLC, as described in Section 3.8.2, at regular time intervals.

### 3.13. Determination of Catalase Activity in Monascus Mycelium during Fermentation Process

Two grams of wet *Monascus* mycelium treated with blue light or kept in darkness were added to 18 mL 0.2 mol mL^−1^, pH 7.8 phosphate buffer from fermentation on day 3. Catalase activity of 10% tissue homogenate was determined by Catalase Assay Kit (Catalog Number CAT100 produced by Sigma-Aldrich) after cells were broken up by ultrasonic cell disruptor. One unit of catalase will decompose 1.0 micromole of hydrogen peroxide to oxygen and water per min at pH 7.0 at 25 °C.

## 4. Conclusions

This study examined the effects of blue light on the growth and pigment yield of *Monascus* in liquid fermentation. We also explored the mechanism behind these effects at the physiological level. Our results showed that *Monascus* pigment content and mycelium cell wall composition changed drastically under blue-light irradiation. Due to the presence of photoreceptors, such as WC-1 and WC-2, *Monascus* mycelium cell permeability and pigment yield were affected in *Monascus* cultured under blue light. Finally, the citrinin yield of *Monascus* under blue light was 50% lower than that cultured in darkness, suggesting that blue light could contribute to degradation of citrinin and affect the catalase activity in *Monascus* mycelium. However, the effect of blue light on other components of the *Monascus* membrane have not been discussed, and the molecular mechanism of blue light affecting the catalase activity needs to be further studied in detail.

## References

[1] Vendruscolo F., Meinicke Bühler R.M., Cesar de Carvalho J., de Oliveira D., Moritz D.E., Schmidell W., Ninow J.L. (2016). Monascus: A Reality on the Production and Application of Microbial Pigments. Appl. Biochem. Biotechnol..

[2] Singgih M., Julianti E. (2015). Food Colorant from Microorganisms[M]//Beneficial Microorganisms in Food and Nutraceuticals.

[3] Sung J., Shin J.Y., Kim H., Baek G.-H., Yu K.-W., Yeon J., Lee J. (2014). Anti-obesity and Anti-hyperlipidemic Activities of Fermented Coffee with *Monascus ruber* Mycelium by Solid-State Culture of Green Coffee Beans. J. Korean Soc. Food Sci. Nutr..

[4] Kwon C.S. (2014). Effect of *Red Yeast* (*Monascus purpureus*) Rice Supplemented Diet on Lipid Profiles and Antioxidant Activity in Hypercholesterolemic Rats. J. Korean Soc. Food Sci. Nutr..

[5] Hsu L.C., Liang Y.H., Hsu Y.W., Kuo Y.H., Pan T.M. (2013). Anti-inflammatory properties of yellow and orange pigments from *Monascus purpureus* NTU 568. J. Agric. Food Chem..

[6] Rochín-Medina J.J., Gutiérrez-Dorado R., Sánchez-Magaña L.M., Reyes Moreno C. (2015). Enhancement of nutritional properties, and antioxidant and antihypertensive potential of black common bean seeds by optimizing the solid state bioconversion process. Int. J. Food Sci. Nutr..

[7] Zhao G.P., Li Y.Q., Yan J., Cui K.Y. (2016). Antibacterial Characteristics of Orange Pigment Extracted from Monascus Pigments against *Escherichia coli*. Czech J. Food Sci..

[8] Lee C.L., Lin P.Y., Hsu Y.W., Pan T.M. (2015). Monascus-fermented monascin and ankaflavin improve the memory and learning ability in amyloid β-protein intracerebroventricular-infused rat via the suppression of Alzheimer’s disease risk factors. J. Funct. Foods.

[9] Chen C.L., Chang K.Y., Pan T.M. (2016). *Monascus purpureus* NTU 568 fermented product improves memory and learning ability in rats with aluminium-induced Alzheimer’s disease. J. Funct. Foods.

[10] Muro Urista C., Gracida Rodríguez J., Abreu Corona A., Ainhoa Arana C., Alejandro Téllez G. (2016). Pigments from fungi, an opportunity of production for diverse applications. Biologia.

[11] Blanc P.J., Loret M.O., Goma G. (1995). Production of citrinin by various species of *Monascus*. Biotechnol. Lett..

[12] Ali N., Blaszkewicz M., Mohanto N.C., Rahman M., Alim A., Hossain K., Degen G.H. (2015). First results on citrinin biomarkers in urines from rural and urban cohorts in Bangladesh. Mycotoxin Res..

[13] Yang J., Chen Q., Wang W., Hu J., Hu C. (2015). Effect of oxygen supply on Monascus pigments and citrinin production in submerged fermentation. J. Biosci. Bioeng..

[14] Hajjaj H., François J.M., Goma G., Blanc P.I. (2012). Effect of amino acids on red pigments and citrinin production in *Monascus ruber*. J. Food Sci..

[15] Haggblom P., Unestam T. (1979). Blue light inhibits mycotoxin production and increases total lipids and pigmentation in *Alternaria alternate*. Appl. Microbiol. Biotechnol..

[16] Fanelli F., Geisen R., Schmidt-Heydt M., Mule G. (2016). Light regulation of mycotoxin biosynthesis: New perspectives for food safety. World Mycotoxin J..

[17] Fanelli F., Reveglia P., Masi M. (2016). Influence of light on the biosynthesis of ophiobolin A by *Bipolaris maydis*. Nat. Prod. Res..

[18] Röhrig J., Kastner C., Fischer R. (2013). Light inhibits spore germination through phytochrome in *Aspergillus nidulans*. Curr. Genet..

[19] Patakova P. (2013). *Monascus* secondary metabolites: production and biological activity. J. Ind. Microbiol. Biotechnol..

[20] Bühler R.M.M., Müller B.L., Moritz D.E., Vendruscolo F., de Oliveira D., Ninow J.L. (2015). Influence of light intensity on growth and pigment production by *Monascus ruber* in submerged fermentation. Appl. Biochem. Biotechnol..

[21] Chen D., Xue C., Chen M., Wu S., Li Z., Wang C. (2016). Effects of blue light on pigment biosynthesis of *Monascus*. J. Microbiol..

[22] Miyake T., Mori A., Kii T., Okuno T., Usui Y., Sato F., Sammoto H., Watanabe A., Kariyama M. (2005). Light effects on cell development and secondary metabolism in *Monascus*. J. Ind. Microbiol. Biotechnol..

[23] Babitha S., Soccol C.R., Pandey A. (2007). Effect of stress on growth, pigment production and morphology of *Monascus* sp. in solid cultures. J. Basic Microbiol..

[24] Schmidt-Heydt M., Cramer B., Graf I., Lerch S., Humpf H.U., Geisen R. (2012). Wavelength-dependent degradation of ochratoxin and citrinin by light in vitro and in vivo and its implications on *Penicillium*. Toxins.

[25] Hajjaj H., Klaebe A., Goma G., Blanc P.J., Barbier E. (2000). Medium-chain fatty acids affect citrinin production in the filamentous fungus *Monascus ruber*. Appl. Environ. Microbiol..

[26] Zigman S., Reddan J., Schultz J.B., McDaniel T. (1996). Structural and Functional Changes in Catalase Induced by Near-UV Radiation. Photochem. Photobiol..

